# A Fourth Supplementary Catalogue of the Plants of Kentucky

**Published:** 1840-10

**Authors:** C. W. Short

**Affiliations:** Professor of Materia Medica and Medical Botany in the Medical Institute of Louisville


Art. II.—*A Fourth Supplementary Catalogue of the Plants of Kentucky.* By C. W. Short, M. D., Professor of Materia Medica and Medical Botany in the Medical Institute of Louisville.

Having several years ago, published in the Transylvania Journal of Medicine, a catalogue of the phenogamous and filicoid plants, native to and naturalized in this state, so far as they had then been observed by me, I afterwards published, in three subsequent numbers of the same Journal, supplementary catalogues of other species, as they became known to me, either by personal observation, or the reports of others competent to determine them. I now offer a *fourth* supplement to the preceding lists, embracing merely the names of, and a few remarks on, some other plants, which have occurred to my notice in different parts of Kentucky, since the publication of the last. Like the others, this list is arranged alphabetically, without reference to systematic classification.

Ampelopsis cordata, on the banks of the Ohio River.

Aspidium dilatatum.

Aspidium asplenoides.

Acerates angustifolia.

Arabis lyrata, found by Dr. Riddell among the knobs of Greenup county, Ky.

Aronia latifolia, from the same locality, detected by the same botanist.

Anthoxanthum odoratum, (Sweet-scented Vernal grass.) This grass, which imparts such delightful odor to new-mown hay, is becoming gradually naturalized in our meadows.

Adonis autumnalis, (Pheasant's eye.) A showy exotic flower, found in the barrens of Kentucky, where it was introduced from a neighbouring garden.

Allium striatum.

Arenaria serpyllifolia.

Angelica triquinata, barrens of Ky.

Angelica atropurpurea, borders of Rock-Castle River, and other mountainous situations.

Asclepias parviflora. This species, so common in the southern States, has only been observed by me in the wet lands bordering on Green River.

Azalea nudiflora. (Bush Honey-suckle.)

Bumelia tenax, on the rocky banks of Little River, a branch of the Cumberland.

Boltonia glastifolia, swamps around Louisville.

Cyperus filiculmis, Islands of the Ohio.

Cyperus erythrorizos, (Grass nut.) The tubers attached to the roots have very much the taste of the cocoa nut.

Cypripedium candidum, (small white lady-slipper.) This interesting species was first pointed out to me in the barrens of Christian county, by the Rev. Mr. Jones, of Hopkinsville, Kentucky.

Chrysanthemum leucanthemum, (Ox-eye daisy.) A troublesome weed in the Eastern States, which will soon be extensively introduced into the West.

Carex bromoides.

Convolvulus sepium, (Bind-weed.)

Convallaria stellata. I have met with this pretty species only on Corn Island, opposite to Louisville.

Clethra tomentosa. I know this plant, as a native of Kentucky, only through a solitary imperfect specimen gathered by my pupil, the late Dr. Clarendon Peck, among the hills of Licking River.

Desmodium strictum.

Eupatoreum rotundifolium.

Epilobium palustre.

Euchroma pallida, barrens of Kentucky; much less abundant than E. coccinea.

Gaura angustifolia.

Gerardia auriculata, wet lands in the barrens.

Hypericum Virginicum, knobs among the barrens.

Hypericum angulosum, " "

Hypericum nudiflorum, " "

Hieracium Kalmii. This plant, in common with many others, is reputed to possess curative properties in snake bites.

Itea Virginica. This pretty shrub was observed by me for the first time this spring, (1840) among the wet timbered lands bordering on Green River, near Rumsey.

Ilex Canadensis, wet lands of Henderson county.

Ilex prinoides, " " "

Juncus marginatus.

Jussieua grandiflora. This plant, to which so much interest attaches, in consequence of the publication of Dr. Cartwright, in the July number of this Journal, was observed by me in the spring of 1838, in a marsh of Henderson county, Kentucky, ten miles south of the Ohio River. It was rare; but its existence there proved its adaptation to the climate; and if the views lately promulgated as to its health-preserving influences be sustained, it would doubtless be desirable to propagate it extensively in malarious and miasmatic districts.

Lythrum alatum.

Liatris cylindracea.

Leavenworthia aurea.

Leavenworthia uniflora, *Torrey.* Cardamine uniflora, *Mich.* These two little plants occur in common on wet rocks among the barrens. The genus, separated by Torrey from Cardamine, has been very justly dedicated to Dr. Leavenworth, of the United States Army, who has done much towards the elucidation of the botany of Louisiana, Arkansas and Florida, whilst stationed at different posts of the South and West.

Mariscus ovularis.

Plantago pusilla. A diminutive species of plantain, frequent in the pastures of Christian county.

Peplis Americana, common in the poor lands and pastures of Muhlenburg county.

Phlox pilosa. This is distinct from the plant, published under the same name, in a previous catalogue. That is most probably P. aristata, and the present is undoubtedly the genuine P. pilosa of Michaux. It occurs in great abundance in early spring among the barrens; and is a very handsome, low species, with dark purple flowers.

Psoralea congesta,—a new species lately discovered by Dr. Clapp and Mr. Jones,of New Albany, on the Islands of the Ohio River, near that place.

Psoralea latifolia. In thickets among the barrens; rather rare.

Prenanthes Illinoensis, abundant in the barrens of Kentucky, as well as the prairies of Illinois.

Quercus triloba. Observed by Dr. Riddell on the knobs of Greenup county, Ky.

Quercus ilicifolia.

Salix petiolaris, } Two dwarf willows detected by Dr. Clapp
Salix longifolia, } on the Islands of the Ohio River.

Sagittaria lanceolata,

Sisymbrium palustre.

Salvia urticifolia, in the thin oak lands of the barrens.

Styllingia sylvatica, rare—barrens of Kentucky.

Stellaria graminea.

Smyrnium atropurpureum.

Sedum telephoides, first pointed out to us by Dr. Clapp, on the lime-stone cliffs above Utica, on the Indiana shore. It no doubt occurs also in similar situations, on the Kentucky side of the Ohio.

Trillium petiolatum. A species having considerable resemblance to the common T. sessile, but totally distinct; barrens of Ky.

Trichostema brachiata, barrens.

Tripsacum dactyloides, (Gama grass.) A luxuriant grass to which public attention was called a few years since, as an excellent article of provender; a character which further experience has proved it not to deserve. It occurs, as a native, among the grasses of the barrens; and has been introduced into different parts of the state.

Taxodium distichum, (Cypress tree.) This truly valuable timber tree is met with abundantly in the lakes and lagoons of all the counties bordering on the Ohio river, in the West of Kentucky; where the peculiar excrescences called "Cypress Knees" form obstacles in the way of crossing the water-courses.

Verbena spuria, roadsides in the barrens, in common with V. angustifolia.

Wistaria frutescens. A flowering pea-vine, now common in gardens and shrubberies; abundant on the banks of Little River in the West of Kentucky.

Xanthium spinosum. A pestiferous species of cockle-bur, which, it is to be feared, will become extensively naturalized. As yet, I have only met with it on the commons of Portland, below the falls of the Ohio.

Yucca filamentosa, (Adam's thread, Bear-grass, &c.) A showy and ornamental plant, frequent in gardens; and which I am informed by the Rev. Mr. Jones, of Hopkinsville, grows abundantly on the Cumberland mountains, in the S. E. corner of Kentucky. An opinion is entertained by some, that the stream which enters the Ohio River at Louisville, derives its its name from this plant, which is supposed to have once grown on its banks. This is most probably erroneous; and it is more likely that the name of the creek was taken from some of those rank and luxuriant grasses, so common in similar alluvions, as Panicum crus-galli, and others.

*August*, 1840.

